# Dissection of the Genetic Architecture for Quantities of Gliadins Fractions in Wheat (*Triticum aestivum* L.)

**DOI:** 10.3389/fpls.2022.826909

**Published:** 2022-03-24

**Authors:** Zhengfu Zhou, Shenghui Geng, Huiyue Guan, Congcong Liu, Maomao Qin, Wenxu Li, Xia Shi, Ziju Dai, Wen Yao, Zhensheng Lei, Zhengqing Wu, Jinna Hou

**Affiliations:** ^1^Henan Institute of Crop Molecular Breeding, Henan Academy of Agricultural Sciences, Zhengzhou, China; ^2^School of Agricultural Sciences, Zhengzhou University, Zhengzhou, China; ^3^National Key Laboratory of Wheat and Maize Crop Science, Henan Agricultural University, Zhengzhou, China

**Keywords:** QTL mapping, gliadin content, KASP marker, wheat, end-use quality

## Abstract

Gliadin is a group of grain storage proteins that confers extensibility/viscosity to the dough and are vital to end-use quality in wheat. Moreover, gliadins are one of the important components for nutritional quality because they contain the nutritional unprofitable epitopes that cause chronic immune-mediated intestinal disorder in genetically susceptible individuals designated celiac disease (CD). The main genetic loci encoding the gliadins were revealed by previous studies; however, the genes related to the content of gliadins and their fractions were less elucidated. To illustrate the genetic basis of the content of gliadins and their fractions comprehensively, a recombinant inbred line (RIL) population that consisted of 196 lines was constructed from the two parents, Luozhen No.1 and Zhengyumai 9987. Quantitative trait loci (QTL) controlling the content of total gliadins and their fractions (ω-, α-, and γ-gliadin) were screened genome-widely under four environments across 2 years. Totally, thirty QTL which explained 1.97–12.83% of the phenotypic variation were detected to be distributed on 17 chromosomes and they were gathered into 12 clusters. One hundred and one pairs of epistatic QTL (E-QTL) were revealed, among which five were involved with the total gliadins and its fractions content QTL located on chromosome 1AS, 1DS, 4DS, 1DL, and 6AS. Three Kompetitive Allele-Specific PCR (KASP) markers were developed from three major QTL clusters located on chromosomes 6A, 6D, and 7D, respectively. The present research not only dissects the genetic loci for improving the content of gliadins and their three fractions, but may also contribute to marker-assisted selection of varieties with appropriate gliadin fractions content for end-use quality and health benefit at the early developmental stages and early breeding generations.

## Introduction

Common wheat, which is one of the most important staple food crops, is mainly grown in temperate regions where most of the global population is distributed. Its adaptability to local climate conditions as well as its unique gluten characteristics makes it suitable for the production of diverse food products. The unique properties of wheat gluten are mainly due to two types of storage proteins, monomeric gliadins and polymeric glutenins, which are responsible for the extensibility/viscosity and strength/elasticity of the dough, respectively.

Gliadins are soluble in 70% ethanol, which distinguishes them from glutenins, and account for about 50–60% of the total gluten content (Tatham and Shewry, 2012). They can be classified as α/β-, γ-, and ω-gliadins according to their mobility during acid-polyacrylamide gel electrophoresis, with α-gliadins migrating fastest and ω-gliadins migrating slowest through the gel (Metakovsky et al., 1993; Qi et al., 2006). The α/β-, γ-, and ω-gliadin fractions represent 55%, 30%, and 15% of the total gliadin content, respectively (Zillman and Bushuk, 1979; Wujun et al., 2019). The molecular structure of gliadins has been revealed (Urade et al., 2018). A signal peptide near the N-terminal is common among the α-, γ-, and ω-gliadins, but the gliadin types are otherwise structurally distinct. More specifically, α-gliadins contain an N-terminal domain I, a repetitive domain, two polyglutamine domains, and two unique domains (Anderson and Greene, 1997; Qi et al., 2006; Zhang et al., 2015). The γ-gliadins consist of an N-terminal non-repetitive domain, a repetitive domain, a non-repetitive domain, a polyglutamine region, and a C-terminal non-repetitive domain (Anderson and Hsia, 2001; Paris et al., 2021) and ω-gliadins contain N- and C-terminal domains and a repetitive domain (Hsia and Anderson, 2001; Anderson et al., 2009). Most α- and γ-gliadins comprise six and eight cysteine residues, respectively, which form intramolecular disulfide bonds that limit the establishment of extensive protein networks (Anderson and Hsia, 2001; Khatkar et al., 2002). However, ω-gliadins rarely contain cysteines and they might interfere with or modify the interactions among glutens, thereby affecting the viscoelasticity of gluten (Khatkar et al., 2002). Gliadins have various effects on rheological properties. The rank order for the weakening effects of the gliadin fractions are ω-1 > ω-2 ≈ α- ≈ β- > γ- as determined using a mixograph, whereas it is γ- > α- ≈ β- ≈ ω-2 ≈ ω-1 according to an extensograph (Fido et al., 1997; Khatkar et al., 2002). Other gliadin compositions also influence the processing quality of gluten. Eliminating ω-5 gliadin reportedly enhances the mixing time and the mixing tolerance of flour (Altenbach et al., 2014). Other gliadin fractions can decrease the mixing time, peak resistance, maximum resistance to extension, loaf height, and dough strength, while increasing the resistance breakdown, extensibility, and loaf volume (Fido et al., 1997; Uthayakumaran et al., 2001).

The gliadin content is an important factor influencing the end-use quality of wheat flour, but it also affects the nutritional properties of the flour. Gliadins contain specific epitopes that can cause celiac disease (CD) (Scherf et al., 2016), which is a chronic immune-mediated intestinal disorder triggered by gluten proteins (mainly gliadins) in genetically susceptible individuals (Sollid, 2000; Ting et al., 2020). These epitopes are rich in proline and glutamine, which can bind to specific human leucocyte antigen class II proteins (DQ2.2, DQ2.5, DQ8, and DQ8.5) in human T-cells, resulting in inflammation and damage to the intestinal mucosa (Vader et al., 2002). All gliadin fractions (α/β-, γ-, and ω-gliadins) induce CD, but α-gliadin is the most potent inducer because it contains the most toxic epitopes derived from a 33-mer peptide sequence (LQLQPFPQPQLPYPQPQLPYPQPQLPYPQPQPF) (Qiao et al., 2005; Camarca et al., 2009). Additionally, α2-gliadin comprises six partially overlapping highly immunogenic T-cell epitopes resulting from the 33-mer peptide sequences (Ozuna et al., 2015). Through RNA interference (RNAi) and ion beam mutagenesis, wheat lines with a significant decrease in the DQ2 and DQ8 CD-related α-gliadin content were obtained; the gluten extracted from these lines failed to elicit T-cell responses *in vitro* (Gil-Humanes et al., 2010; Wang et al., 2017). However, there is currently a lack of gliadin-free wheat varieties bred using a traditional breeding strategy.

Previous studies on common wheat identified the major genetic loci for gliadins (*Gli-1* and *Gli-2*) in the homologous regions of the short arms on chromosomes 1 and 6, with *Gli-1* encoding all of the ω-gliadins and many of the γ-gliadins and *Gli-2* encoding all of the α-gliadins, many of the β-gliadins, and some of the γ-gliadins (Sabelli and Shewry, 1991; Redaelli et al., 1994; Wang et al., 2020). Other loci with minor effects on the gliadin content (mainly ω-gliadins) have also been detected, including *Gli-3*, *Gli-5*, *Gli-6*, and *Gli-7* (Galili and Feldman, 1984; Pogna et al., 1993; Hassani et al., 2010). Multiple copies of tandemly repeated gliadin genes have been identified, with a high sequence similarity among copies. Earlier research revealed that there are usually 25–35 copies of α-gliadin genes, but there may be as many as 150 copies (Harberd et al., 1985; Anderson et al., 1997; Huo et al., 2017), whereas there are 15–40 copies of γ-gliadin genes (Sabelli and Shewry, 1991). However, some of these copies are pseudogenes or truncated genes that do not contribute to the total gliadin content. In Xiaoyan 81, among the 52 transcribed gliadin genes, 42 comprise an intact coding sequence, and at least 38 encode proteins that accumulate in mature grains (Wang et al., 2017). Due to the rapid evolution and artificial selection during domestication, the genomes of wheat cultivars (accessions) have accumulated extensive sequence variations in the repetitive region of gliadin genes and allelic gene expression variants (Shewry et al., 2003; Qi et al., 2006; Huo et al., 2019). Therefore, elucidating the functional variation among gliadin alleles, which will be useful for developing wheat varieties with decreased gliadin contents and health benefits for humans, is a major challenge. Quantitative trait locus (QTL) mapping is an effective strategy. Although QTLs related to the gliadin content have been detected on chromosomes 1B, 2A, 4A, 5A, 6B, 6D, 7A, and 7B (Charmet et al., 2005; Zhao et al., 2017), many related QTLs remain to be characterized in diverse cultivars.

In the present study, a recombinant inbred line (RIL) population was constructed from a cross between Luozhen No. 1 and Zhengyumai 9987. A total of 30 QTLs related to gliadin contents (total as well as α/β-, γ-, and ω-gliadin fractions) were detected on 17 chromosomes at two study locations across 2 years. These QTLs explained 1.97%–12.83% of the phenotypic variation, and the logarithm of odds (LOD) value was 2.50–8.41. The 30 QTLs were grouped in 12 clusters in the physical map. Three Kompetitive Allele-Specific PCR (KASP) markers were developed from three QTL clusters on chromosomes 6A, 6D, and 7D. Additionally, 101 pairs of epistatic QTL (E-QTL) were revealed, among which five were associated with the QTL on chromosomes 1AS, 1DS, 4DS, 1DL, and 6AS. This study identified the genetic loci influencing the gliadin fraction contents. Furthermore, the KASP markers developed from the QTL clusters may facilitate the identification of elite germplasms and the marker-assisted selection of wheat lines with modulated gliadin contents.

## Materials and Methods

### Materials and Field Experiments

A population comprising 196 RILs was constructed *via* the hybridization between Luozhen No. 1 and Zhengyumai 9987 (Zhou et al., 2020b), which were provided by the Luohe Academy of Agricultural Sciences and the Youbang Crop Breeding Institute in Zhengzhou, respectively. The two parents varied in terms of agricultural and quality-related traits, especially the gliadin composition and content.

The RIL population was grown at the field experimental station of the Henan Academy of Agricultural Sciences in Yuanyang (E113°97′, N35°05′) and Shangqiu (E115°65′, N34°45′), Henan province, China, during the 2018–2019 and 2019–2020 growing seasons (E1, E2, E3, and E4 corresponded to 2018–2019 in Yuanyang, 2018–2019 in Shangqiu, 2019–2020 in Yuanyang, and 2019–2020 in Shangqiu, respectively). The seeds of each RIL were sown using a single-seed precision sowing method and arranged according to a randomized block design. Each line was grown in a plot consisting of two rows (2 m × 0.3 m), with 10 cm distance between adjacent plants. Seeds were sown during the most suitable period in mid-October, and the resulting plants were harvested in May of the following year. Fertilizers were applied and disease and pest control measures were implemented according to local management practices during the wheat plant developmental period.

The association population was consisted of 207 previously reported wheat varieties collected worldwide (Zhou et al., 2020a,^2021^). Plants were grown in 2017 and 2018 at two locations (E5, E6, and E7 corresponded to 2017 in Yuanyang, 2018 in Yuanyang, and 2018 in Shangqiu, respectively).

### Gliadin Extraction

Gliadins were extracted from 45 mg whole-meal flour, as previously described (Lookhart et al., 1993), with minor modifications. Briefly, 1 ml of 70% ethanol was added to the whole-meal flour. The resulting solution was thoroughly mixed in a vertical mixer for 1 h at room temperature and then centrifuged (12,000 rpm for 10 min). The supernatant was collected and filtered through nylon film with a diameter of 0.45 μm. Gliadins were present in the filtrate. Two biological replicates were prepared for each RIL for the gliadin extraction and subsequent gliadin content measurement.

### Gliadin Content Measurement

A reversed-phase high-performance liquid chromatography (RP-HPLC) system (Waters E2695+2998DAD; Waters Corporation, Milford, MA, United States) with the Vydac 218TP C18 HPLC column (250 mm × 4.6 mm) was used for analyzing the gliadins in 200 μl extracts. The parameters were set as follows: elution flow rate, 0.8 ml/min; elution gradient, 0–30 min when 90% eluent A (0.06% TFA solution in ddH_2_O, v/v) was linearly decreased to 64 and 21% eluent B (0.05% TFA solution in acetonitrile) was increased to 36%, 30–51 min when eluent A was linearly decreased to 53.5% and eluent B was increased to 46.5%, 51–56 min when eluent A was increased to 79% and eluent B was decreased to 21%, and 56–60 min when eluent A and eluent B were maintained at 79 and 21%, respectively. The column temperature was set at 60°C and the injection volume was 10 μl. The total gliadin content was calculated according to the chromatogram peak area. The content of each gliadin fraction was calculated according to the area of the corresponding peaks as follows:


$$Y\,u=\frac{T\,u\times 100}{M\,\left(1-X\right)}$$

where Yu (10^6^ AU/mg) is the content of an individual fraction; *M* (mg) is the weight of the sample used for the gliadin extraction; *X* (%)is the water content of the wheat flour determined using a near-infrared spectrum analyzer; *Tu* (AU) is the peak area of an individual fraction, and ×100 is 10 μl of a 1 ml extract analyzed by RP-HPLC.

### Statistical Analysis

Various parameters of the phenotypic data (e.g., range, average, standard deviation, coefficient of variation, kurtosis, and skewness) were analyzed using Microsoft Excel with the “Analysis ToolPak–VBA” add-in. The IBM SPSS Statistics 22 software was used for analyzing variations and correlations. The best linear unbiased prediction (BLUP) and broad-sense heritability evaluation of each trait in different environments were performed using the “Lme4” package in R. The figures presenting the phenotypic distribution and histograms were made using the Origin 2017 software.

### Quantitative Trait Locus Mapping and Detection of Epistatic Quantitative Trait Locus for Gliadins

A genetic map constructed using 8,518 SLAF markers distributed across all 21 wheat chromosomes and covering 3,140.54 cm (Zhou et al., 2020b) was used for QTL mapping. The QTL mapping was conducted according to the composite interval mapping (CIM) method using the “QTL. gCIMapping. GUI” software package in R. The parameters were set as follows: LOD score = 2.5; random model and walking speed for genome-wide scanning = 1 cM. The additive-by-additive E-QTL was analyzed using the QTL IciMapping software. More specifically, the ICIM-EPI method was used with the following mapping parameters: step (cM), 25; probability in stepwise regression, 0.0001; and LOD threshold, 1.000 (Li et al., 2007, 2008).

### Physical Map Construction

The SLAF markers on the linkage map were aligned and mapped to the corresponding positions on the physical map of the Chinese Spring reference genome (IWGSC RefSeq v1.1) (International Wheat Genome Sequencing, 2018) using the default parameters of the Burrows–Wheeler Aligner software (Li and Durbin, 2009). The MapChart program was used to present QTL on the physical map.

### Kompetitive Allele-Specific PCR Marker Development

Genomic sequences 100 bp long in the 5′ and 3′ strands surrounding the target SNP were extracted. Two allele-specific primers were designed to include the FAM (5′-TGAAGGTGACCAAGTTCATGCT-3′) or the HEX (5′-GAAGGTCGGAGTCAACGGATT-3′) sequence at the 5′ end and the target SNP was anchored at the 3′ end. The sequences from which the target SNPs were derived were used to identify homologous sequences *via* a BLAST search of the EnsemblPlants database^1^ using default parameters and 5–8 sequences with the highest homology were selected. These sequences were aligned and the conserved regions were used for designing allele-specific primer pairs. The PCR was performed using the KASP Assay mixture and the Bio-Rad CFX Maestro system was used for fluorescence detection and data analysis.

### Identification of Differentially Expressed Genes in Three Major Quantitative Trait Locus Regions

Developing grains at 5, 10, 15, 20, 25, and 30 days after pollination (DAP) were collected from the two parents (Luozhen No. 1 and Zhengyumai 9987). Total RNA was extracted from the grain samples, with three replicates each. The integrity of the RNA was evaluated using the Agilent 4200 TapeStation system, and the quality of the RNA was assessed using the Qubit 3.0 Fluorometer (Thermo Fisher Scientific, Waltham, MA, United States). High-quality RNA samples were used for constructing RNA-seq libraries, which were analyzed on an Illumina system. The clean RNA-seq data were assembled based on the Chinese Spring reference genome sequence (IWGSC RefSeq v1.1). Gene expression levels were calculated in terms of the fragments per kilobase of transcript per million fragments mapped (FPKM) value. The differentially expressed genes were screened using the following criteria: false discovery rate <0.01 and expression level difference >2 times.

## Results

### Phenotypic Variation in the Recombinant Inbred Line Population

**FIGURE 1:**
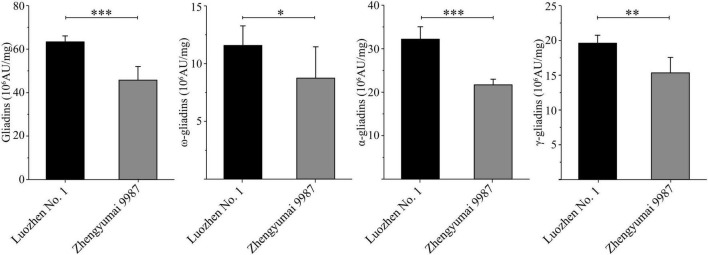
Phenotypic diversity in the gliadin contents between the two parents of the recombinant inbred line (RIL) population. Data are presented as the average gliadin contents (total and individual fractions) at two locations across 2 years. The fractions are labeled on the y-axis. Column heights reflect the gliadin contents. The significance of the differences between the two parents was evaluated by *t*-test: ^∗^, ^∗∗^, and ^∗∗∗^ represent *P* < 0.05, *P* < 0.01, and *P* < 0.001, respectively.

The two parents and the RIL population were grown at two locations across 2 years. The total gliadin and α-, γ-, and ω-gliadin contents, which were determined on the basis of an RP-HPLC analysis, were respectively 63.34, 32.17, 19.61, and 11.57 (10^6^ AU/mg) for Luozhen No. 1 and 45.75, 21.67, 15.33, and 8.74 (10^6^ AU/mg) for Zhengyumai 9987. The gliadin contents were significantly higher in Luozhen No. 1 than in Zhengyumai 9987 (*P* < 0.05, Figure 1 and Supplementary Table 1). The differences were greatest for the total gliadin and α-gliadin contents (*P* < 0.001, Figure 1 and Supplementary Table 1).

**FIGURE 2:**
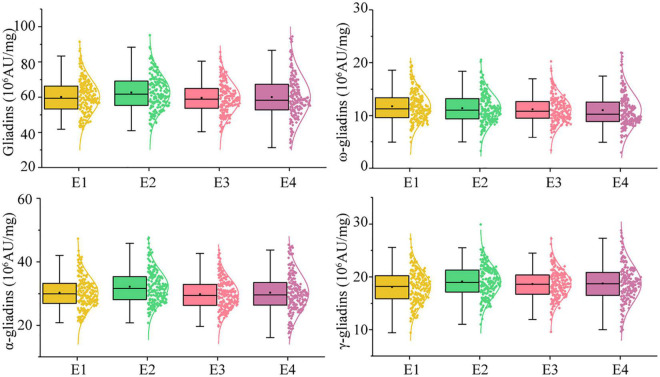
Phenotypic distribution profiles of gliadin contents in the RIL population at two locations across 2 years. The scattered dots with different colors represent the gliadin contents (total and individual fractions) of each line. The corresponding growth conditions are indicated on the x-axis and the values of each dot are indicated on the y-axis. The curve represents the probability density of the raw data. The boxplot indicates the median value (horizontal line) as well as the lower and upper quartiles of the data. The whiskers of the boxplot define the data limits. E1, E2, E3, and E4 correspond to 2018–2019 in Yuanyang, 2018–2019 in Shangqiu, 2019–2020 in Yuanyang, and 2019–2020 in Shangqiu, respectively.

In the RIL population, the total gliadin and α-, γ-, and ω-gliadin contents varied substantially in all the environments (Supplementary Table 2 and Figure 2). The average total gliadin content was 41.70–88.65 (10^6^ AU/mg) and the α-, γ-, and ω-gliadin contents were in the range of 21.46–43.29, 9.93–27.89, and 4.23–18.73 (10^6^ AU/mg), respectively. Similar to most quality-related traits, the gliadin contents (total and individual fractions) in the RIL population exhibited a continuous variation and a normal distribution (Figure 2 and Supplementary Table 2).

The contributions of various factors to the observed variations in the gliadin contents (total and individual fractions) were examined *via* an ANOVA. Generally, the variations were due to the genotypes ($\sigma_{G}^{2}$), the environments ($\sigma_{E}^{2}$), and the genotype × environment interactions ($\sigma_{G\,E}^{2}$) (*p* < 0.001), with the exception of the variation due to errors ($\sigma_{e}^{2}$) (Table 1). The broad-sense heritability (*H*^2^) exceeded 0.80 (Table 1), implying that genotypes were the most important factors controlling gliadin contents (total and individual fractions).

**TABLE 1:** Analysis of phenotypic variance components for gliadin fractions in the recombinant inbred line (RIL) population across 2 years.

	ANOVA*^a^*				
Trait	σ${}_{G}^{2}$	σ${}_{E}^{2}$	σ${}_{G\,E}^{2}$	σ${}_{e}^{2}$	*H* ^2b^
Glia	630.83***	753.91***	74.97***	3.83	0. 85
ω-gli	45.04***	39.45***	6.95***	0.37	0.81
α-gli	168.09***	425.68***	19.84***	1.74	0.86
γ-gli	59.73***	72.81***	6.01***	0.45	0.86

^a^Analysis of variance of individual traits. Variance contributed by the genotypes ($\sigma_{G}^{2}$), environments ($\sigma_{E}^{2}$), genotype × environment interactions ($\sigma_{G\,E}^{2}$), and errors ($\sigma_{e}^{2}$). ***Variances contributed by the genotypes, environments, and genotype × environment interactions were significant (P < 0.001).

*^b^Broad-sense heritability (H^2^) of each trait in the RIL population at two locations across 2 years.*

### Genetic Loci for the Total Gliadin Content

**FIGURE 3:**
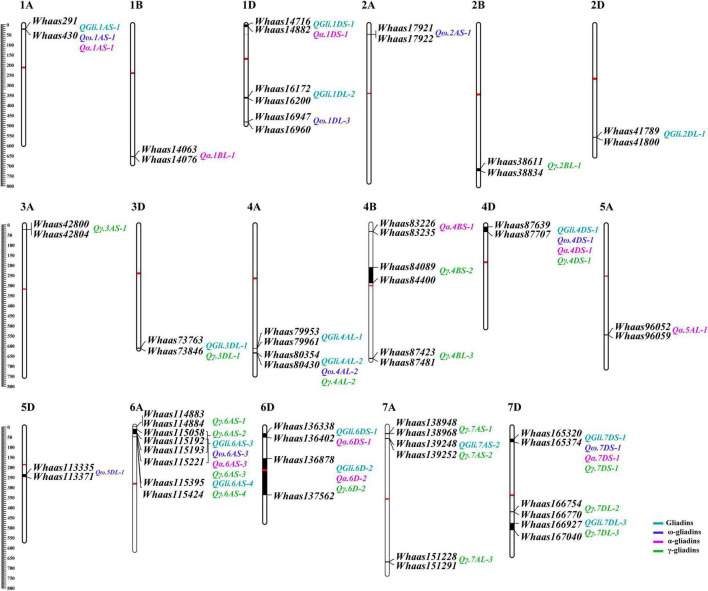
The quantitative trait loci (QTL) for gliadin contents (total and individual fractions) distributed on the physical map. The QTL represented by black bars are mapped to physical positions on chromosomes and the associated markers are provided to the right of each chromosome. The physical positions are indicated to the left of the first chromosome in each row. QTLs associated with different traits are assigned different colors (legends in the lower right corner). The centromere of each chromosome is indicated by a red bar.

A previously described genetic map consisting of 8,518 SNPs and covering 3,140.54 cM with chromosome lengths ranging from 94.33 (3D) to 207.17 cM (7A) (Zhou et al., 2020b), was used for screening the genetic loci related to the gliadin contents (total and individual fractions) in the RIL population. Regarding the total gliadin content, 15 QTLs were detected on chromosomes 1A, 1D, 2D, 3D, 4A, 4D, 6A, 6D, 7A, and 7D (Figure 3, Supplementary Table 3, and Supplementary Figure 1). More specifically, chromosomes 1D, 4A, 6A, 6D, and 7D contained two QTLs each, whereas chromosomes 1A, 2D, 3D, 4D, and 7A had only one QTL (Figure 3, Supplementary Table 3, and Supplementary Figure 1). The number of QTLs detected varied among environments (Figure 3, Supplementary Table 3, and Supplementary Figure 1), with the least QTLs (i.e., two) detected in E3 (2020 in Yuanyang). In contrast, eight QTLs were detected when the BLUP value was applied as an independent environment (Figure 3, Supplementary Table 3, and Supplementary Figure 1). The QTL accounted for 2.98–12.07% of the phenotypic variation, with an LOD value ranging from 2.75 to 8.14. Two QTLs (*QGli.6D-2* and *QGli.7DS-1*) that accounted for 5.48%–12.07% of the phenotypic variation (LOD = 3.34–8.14) were detected in three or more environments (BLUP included) and were considered as the major genetic loci for the total gliadin content.

### Quantitative Trait Locus Mapping for Three Gliadin Fraction Contents

A total of 36 QTLs were detected for gliadin fraction contents, of which 8, 10, and 18 QTLs were related to the ω-, α-, and γ-gliadin contents, respectively. The QTLs for the ω-gliadin content were distributed on chromosomes 1A, 1D, 2A, 4A, 4D, 5D, 6A, and 7D, with an LOD value of 2.55–4.39. The QTLs with the largest effect on the ω-gliadin content (*Qω.1AS-1*), which was specifically detected in E2, explained 8.66% of the phenotypic variation (Figure 3, Supplementary Table 3, and Supplementary Figure 2). However, *Qω.5DL-1* and *Qω.6AS-3*, which explained 5%–6.26% and 5.53%–7.2% of the phenotypic variation, respectively (LOD = 2.55–3.55 and 2.85–4.39, respectively), were designated as the major genetic loci for the ω-gliadin content because they were detected in three or more environments. Similarly, *Qα.1DS-1* and *Qα.7DS-1*, which explained 4.01%–11.2% and 5.65%–12.83% of the phenotypic variation, respectively, were considered as the major QTLs for the α-gliadin content (Figure 3, Supplementary Table 3, and Supplementary Figure 3), and they were revealed to have the largest effect on the α-gliadin content. Among the 18 γ-gliadin-related QTLs, *Qγ.4AL-2*, *Qγ.6D-2*, and *Qγ.7DS-1* were detected in three or more environments (BLUP included) and were identified as the major QTL (Figure 3, Supplementary Table 3, and Supplementary Figure 4). The individual QTLs explained 3.02%–10.12% of the phenotypic variation (Figure 3, Supplementary Table 3, and Supplementary Figure 4).

### Analysis of the Physical Positions of Quantitative Trait Locus

**FIGURE 4:**
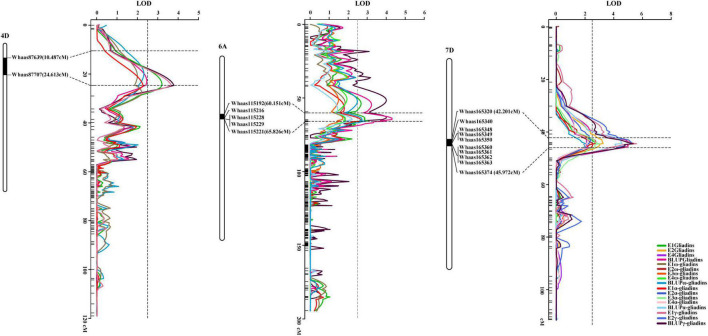
Three major QTL clusters for gliadin contents (total and individual fractions). The QTL clusters for all investigated traits on chromosomes 4D, 6A, and 7D are presented. The QTLs screened in the genetic map and their relative physical positions are integrated in each figure. The genetic distance for each major QTL is presented in terms of centimorgans (cM). The corresponding physical positions are indicated by black bars, with the associated markers labeled to the right of the chromosomes, which are presented as white columns. The curves with various colors represent different traits (legends in the lower right corner). Horizontal dotted lines indicate the confidence intervals of the QTL, whereas the vertical dotted line represents an LOD value of 2.5.

Interestingly, when the QTLs detected in the present study were mapped to the physical map of the Chinese Spring reference genome according to their tightly linked SLAF tag sequence, the genetic loci for various traits were clustered in the same genomic regions. Thirty genomic regions distributed on 17 chromosomes in the physical map were determined to influence the gliadin contents (total and individual fractions). Of these genomic regions, six were associated with two traits, whereas three were associated with three or four traits (Figure 3 and Supplementary Table 4). Analyses of specific chromosome regions indicated that 1AS-1 affected the total gliadin, ω-gliadin, and α-gliadin contents; 4AL-2 contributed to the total gliadin, ω-gliadin, and γ-gliadin contents; and 6D-2 controlled the total gliadin, α-gliadin, and γ-gliadin contents (Figure 4 and Supplementary Table 4). Furthermore, three chromosome regions (4DS-1, 6AS-3, and 7DS-1) included genetic loci for all four analyzed traits (i.e., major QTL clusters) (Figures 3, 4 and Supplementary Table 4). The QTLs in these three regions accounted for 4.68%–8.02%, 5.07%–9.16%, and 4.93%–12.07% of the phenotypic variation, with an LOD value of 3.27–5.69, 2.85–6.47, and 3.06–8.41 (Figures 3, 4 and Supplementary Table 4).

### Detection of Epistatic Quantitative Trait Locus in Multiple Environments

**FIGURE 5:**
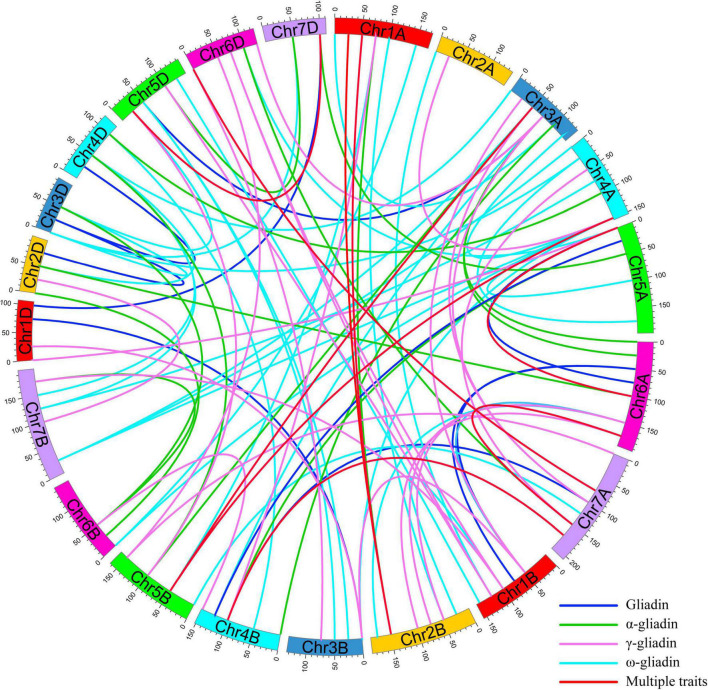
E-QTL for the gliadin contents (total and individual fractions) in the RIL population. The E-QTL interval pairs are linked by curves. Various colors represent the E-QTL for different gliadin fractions (legends in the lower right corner). Colored bars represent chromosomes and the genetic positions are indicated on the outer part of the circular chromosomes.

To interpret the epistatic effects of different genetic loci, 101 pairs of significant E-QTL were detected for the gliadin contents (total and individual fractions) in the RIL population in four environments (Figure 5 and Supplementary Table 5). More specifically, 20, 34, 22, and 25 E-QTLs were detected for total gliadin and ω-, α-, and γ-gliadin contents, respectively. These E-QTLs were estimated to explain 3.27%–10.72% of the phenotypic variation, with an LOD value of 2.50–4.13 (Supplementary Table 5). Among these E-QTLs, two for the ω- and γ-gliadin contents were detected in three environments, whereas three for the total gliadin as well as the α- and γ-gliadin contents were detected in two environments (Supplementary Table 5). Five QTL intervals on chromosomes 1AS, 1DS, 4DS, 1DL, and 6AS contributed to the E-QTL pairs.

### Development of Kompetitive Allele-Specific PCR Markers for Three Quantitative Trait Locus Clusters

**FIGURE 6:**
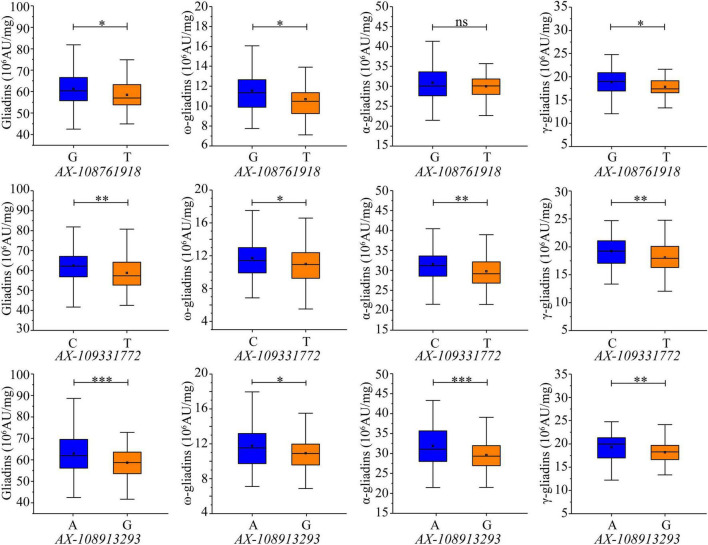
Effects of KASP markers on gliadin content variations in the RIL population. Genotypes were detected according to the KASP markers developed from the SNPs AX-108761918, AX-109331772, and AX-108913293, which are labeled under each chart. The y-axis indicates the gliadin fraction contents. Significant differences among the various genotypes are indicated by ^∗^, ^∗∗^, and ^∗∗∗^, which correspond to *P* < 0.05, *P* < 0.01, and *P* < 0.001, respectively; ns indicates a lack of a significant difference between the two genotypes.

The 660K chip SNPs closest to the SLAF markers tightly linked to the QTL clusters were selected for developing KASP markers. Two KASP markers were developed from the SNPs (AX-109331772 and AX-108913293) closest to the SLAF markers *Whaas115221* and *Whaas165374*, which were associated with the major QTL clusters in 6AS-3 and 7DS-1, respectively (Supplementary Table 6). Although KASP markers were not developed for the major QTL cluster in 4DS-1, a third KASP marker was developed from AX-108761918, which was closest to the SLAF marker (*Whaas136878*) tightly linked to the QTL cluster in 6D-2 related to the total gliadin as well as the α- and γ-gliadin contents. All three KASP markers were able to distinguish allelic diversity. The contribution of each allele was evaluated in the RIL population (Supplementary Table 7). For KASP markers AX-108761918 and AX-109331772, the increasing alleles included the SNP “G” or “C” from the Luozhen No. 1 genome; however, the increasing allele for AX-108913293 included the SNP “A” from the Zhengyumai 9987 genome (Supplementary Table 7). Additionally, AX-108761918-G increased the total gliadin, ω-gliadin, and γ-gliadin contents by 4.74%, 7.86%, and 5.86%, respectively, in the RIL population (Figure 6 and Supplementary Table 7). The analysis of the RIL population also revealed that AX-109331772-C significantly increased the total gliadin, ω-gliadin, α-gliadin, and γ-gliadin contents by 6.22%, 6.54%, 5.95%, and 6.47%, respectively (Figure 6 and Supplementary Table 7), whereas AX-108913293-A significantly increased the total gliadin, ω-gliadin, α-gliadin, and γ-gliadin contents by 7.23%, 7.62%, 7.71%, and 6.2%, respectively (Figure 6 and Supplementary Table 6). In addition to the observed effects in the RIL population, AX-108913293-A also significantly increased the ω-gliadin content (*P* < 0.01) in 202 wheat accessions collected worldwide (Zhou et al., 2020a) (Supplementary Figure 5 and Supplementary Table 8).

### Additive Effect of Increasing Alleles on Gliadin Fraction Contents

**FIGURE 7:**
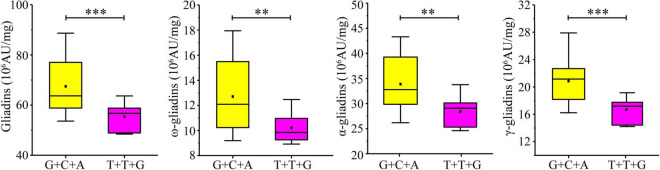
Additive effect of three KASP markers on gliadin content variations in the RIL population. Four groups of allelic variants were compared in terms of the total gliadin content. The nucleotide combinations of each line are labeled on the x-axis, with the order of the genotypes detected on the basis of the significant SNPs AX-108761918, AX-109331772, and AX-108913293. ^∗∗∗^ and ^∗∗^ indicate significant differences at the *P* < 0.001 and *P* < 0.01 levels, respectively.

Considering the significant influence of the three developed KASP markers on the gliadin contents (total and individual fractions), the additive effect of the three increasing alleles was analyzed in the RIL population. A total of 27 lines in the RIL population contained increasing alleles for all three loci (AX-108761918-G + AX-109331772-C + AX-108913293-A), whereas six lines contained decreasing alleles (AX-108761918-T + AX-109331772-T + AX-108913293-G). The increasing alleles were associated with significant increases in the gliadin contents (total and individual fractions). More specifically, the total gliadin, ω-gliadin, α-gliadin, and γ-gliadin contents were respectively 21.89%, 24.51%, 19.17%, and 24.99% higher in the lines with increasing alleles than in the lines with decreasing alleles (Figure 7).

### Differentially Expressed Genes in Three Major Quantitative Trait Locus

The differentially expressed genes in the major QTL on chromosomes 4D, 6A, and 7D were identified. Of the 294 differentially expressed genes in these three major QTL-containing regions, 49, 80, 39, 31, 46, and 49 were detected at 5, 10, 15, 20, 25, and 30 DAP, respectively (Supplementary Table 9). The differentially expressed genes varied among the developmental stages, with 167 detected in only one developmental stage. The annotation of the differentially expressed genes, on the basis of a GO analysis, indicated that they encode diverse proteins, including transcription factors, hydrolases, proteolytic enzymes, RNA polymerases, and histones (Supplementary Table 9). Finally, one gene (*TraesCS4D01G047400*) and two genes (*TraesCS6A01G049100* and *TraesCS6A01G049200*) in the QTL regions on chromosomes 4D and 6A, respectively, were considered as candidate genes related to glutamine synthetase (GS2) and α-gliadin that control the gliadin content (Supplementary Table 9). A previous study demonstrated that GS2-encoding genes are important for plant nitrogen metabolism because of their role in the first step of ammonium assimilation and the conversion to glutamine and grain proteins in wheat (Gadaleta et al., 2011).

## Discussion

### Comparison of the Quantitative Trait Locus Mapping Results Between Studies

Gliadins, which account for the largest fraction of wheat grain proteins, are closely related to the processing and nutritional value of wheat flour. Previous analyses detected a series of QTLs related to the protein contents in a population derived from two parents and in a population comprising natural wheat varieties (accessions) (Table 2) (Prasad et al., 2003; Plessis et al., 2013; Nigro et al., 2019; Lou et al., 2021). The major genetic loci controlling protein contents are on chromosomes 1A, 1B, 2A, 2B, 2D, 3A, 3B, 3D, 4A, 4B, 5A, 5B, 5D, 6B, and 7B (Cui et al., 2016; Nigro et al., 2019; Li et al., 2020; Lou et al., 2021). The QTLs associated with the gliadin content have been mapped to chromosomes 1B, 2A, 3B, 4A, 5A, 7A, and 7B (Plessis et al., 2013; Zhao et al., 2017) (Table 2). In the present study, 30 QTLs for gliadin contents (total and individual fractions) were detected on chromosomes 1AS, 1BL, 1DS, 1DL, 2AS, 2BL, 2DL, 3AS, 3DL, 4AL, 4BS, 4BL, 4DS, 5AL, 5DL, 6AS, 6DS, 7AS, 7AL, 7DS, and 7DL in the RIL population (Figure 3 and Supplementary Tables 3, 4). Our results included all the genetic loci related to the gliadin content detected in previous studies. Additionally, novel genomic regions, including the QTL clusters in 4DS-1 and 7DS-1, that were revealed in this study may be useful for further clarifying the molecular mechanisms underlying gliadin contents (total and individual fractions). However, certain QTLs on chromosome 3B that were identified in previous studies were not detected in the present study, possibly because of the diversity in the environmental conditions and/or genetic backgrounds of the varieties between studies (Wan et al., 2013). Increases in the nitrogen supply result in increased grain gliadin contents because of the enhanced synthesis and accumulation of storage proteins (Godfrey et al., 2010). In contrast, increases in the available sulfur content significantly decrease the gliadin content, while also increasing nitrogen-use efficiency by more than 20% (Yu et al., 2021). The findings of the present study may provide wheat researchers and breeders with useful information for modifying gliadin contents; however, more research is required to elucidate the complex genetic mechanism regulating gliadin contents (total and individual fractions). For example, the QTL cluster in 6D-2 spans the centromere region. Therefore, additional molecular markers are needed to further dissect this QTL cluster.

**TABLE 2:** Overview on common wheat quantitative trait loci (QTL) for gliadins reported in recent literature.

Chr	Cluster	Flanking markers	References
1AS	1AS-1	*Whaas*291 ∼ *Whaas*430	
	*QLGPC.cau*-*1A*	GENE-0412_338	Lou et al., 2021
1BL	1BL-1	*Whaas*14063 ∼ *Whaas*14076	
	*QNGPC.cau*-*1B*	wsnp_RFL_Con_tig3866_4228783	Lou et al., 2021
2AS	2AS-1	*Whaas*17922 ∼ *Whaas*17921	
	*QGpc.ccsu-2A.1*	*Xgwm830*	Prasad et al., 2003
	*QGpc.ccsu-2A.2*	*Xgwm726*	
2BL	2BL-1	*Whaas*38611 ∼ *Whaas*38834	
	*QGpc.ccsu-2B.1*	*Xgwm1249*	Prasad et al., 2003
	*QLGPC.cau*-*2B*	BS00067201_51	Lou et al., 2021
2DL	2DL-1	*Whaas*41789 ∼ *Whaas*41800	
	*QGpc.ccsu-2D.1*	*Xgwm1264*	Prasad et al., 2003
	*QGpc.ccsu-2D.2*	*Xgwm1204*	Prasad et al., 2003
3AS	3AS-1	*Whaas*42800 ∼ *Whaas*42804	
	*QLGPC.cau*-*3A.1*	c67727_66398596	Lou et al., 2021
3DL	3DL-1	*Whaas*73763 ∼ *Whaas*73846	
	*QNGPC.cau*-*3D*	RFL_Contig148_359	Lou et al., 2021
		wPt5506	Plessis et al., 2013
4AL	4AL-1	*Whaas*79961 ∼ *Whaas*79953	
	4AL-2	*Whaas*80354 ∼ *Whaas*80430	
	*QGpc.ccsu-4A.1*	*Xgwm894*	Prasad et al., 2003
	*QGpc.ccsu-4A.2*	*Xgwm397*	Prasad et al., 2003
6AS	6AS-1	*Whaas*114883 ∼ *Whaas*114884	
	6AS-2	*Whaas*115058 ∼ *Whaas*115193	
	6AS-3	*Whaas*115192 ∼ *Whaas*115221	
	6AS-4	*Whaas*115395 ∼ *Whaas*115424	
	*QNGPC.cau*-*6A*	TA003913-0402	Lou et al., 2021
	7AS-1	*Whaas*138948 ∼ *Whaas*138968	
	7AS-2	*Whaas*139248 ∼ *Whaas*139252	
7AS	*QGpc.ccsu-7A.1*	*Xgwm1171*	Prasad et al., 2003
	*QGpc.mgb*-*7A*	IWB65659	Nigro et al., 2019

### Candidate Genes Predicted According to the Previously Revealed Molecular Mechanism Related to the Gliadin Content

The unique gluten property of wheat flour makes it suitable for the production of a wide range of food products. Gliadins, which are responsible for dough extensibility, are exclusive to the Triticeae species. Evolutionary analyses revealed gliadins developed after glutenins and evolved after the speciation of wheat, with α-gliadins identified as the youngest gluten component that is not present in the related species, namely, barley and rye (Huo et al., 2017, 2018). The α-gliadin genes evolved rapidly and independently in the A, B, and D genomes of different wheat species (Huo et al., 2019). In the present study, two α-gliadin genes were annotated and selected the candidate genes (Gadaleta et al., 2011; Huo et al., 2017) in the major QTL on chromosome 6A. These two genes were clustered together, which is consistent with the genomic arrangement of previously characterized gliadin genes. The polymorphisms in the α-gliadin alleles or the diversity in their copy number in the genome likely differentially affect the gliadin content. However, there were no polymorphisms in the two α-gliadin genes in the two parents. Therefore, future research on α-gliadin genes should focus on elucidating the variations in allelic sequences and copy numbers in the target QTL region. Moreover, an epigenetic mechanism might exist under this QTL. As an important mechanism in gene regulation, few studies revealed its function on gliadin content control. Previous research reported that the DNA methylation in the promoter region of the gliadin gene, *TaGli-γ-2.1*, negatively regulates gliadin content in wheat significantly (Zhou et al., 2021). That gave us a clue that the epigenetic mechanism, such as DNA methylation or/and histone modification under the QTL 7DS, not the sequence divergence of the candidate gene, influenced the gliadin content. Furthermore, for the QTL cluster in 6D-2, which stretches over the centromere region, additional molecular markers should be developed to enable further analyses. Regarding the major QTL on chromosome 4D, the GS2 gene (*TraesCS4D01G047400*) was identified as a candidate gene because the encoded protein reportedly enhances nitrogen assimilation and increases the grain storage protein content in durum wheat (Habash et al., 2007; Bernard et al., 2008; Gadaleta et al., 2011; Zheng et al., 2018). An earlier study detected GS2 genes on durum wheat chromosomes 2A, 2B, 4A, and 4B (Gadaleta et al., 2011). In the current study, we revealed the candidate gene in the homologous region on chromosome 4D in common wheat. Although candidate genes were not identified in the QTL region on chromosome 7D, the effect of this locus must be considered. There may be an unreported regulatory mechanism underlying the gliadin content.

### The Results of This Study May Be Helpful for Simultaneous Improvement of the End-Use and Health Benefits Qualities Breeding in Wheat

Gliadins, especially α-gliadins, are potent inducers of the gastrointestinal and non-gastrointestinal inflammation symptoms of CD. Hence, the most effective strategy for preventing CD symptoms in susceptible populations involves the elimination of wheat products from the diet (Biesiekierski et al., 2013; Sharma et al., 2020), but it may be difficult to completely exclude wheat products from the diet for an entire lifetime. Therefore, breeding gliadin-free or reduced-gliadin wheat varieties is a realistic approach to minimize the effects of CD in susceptible populations (Jouanin et al., 2018; Yoosuf and Makharia, 2019). Several studies have used RNAi and CRISPR/Cas9 techniques to produce wheat accessions with fewer or no CD epitopes (Gil Humanes et al., 2008; Gil-Humanes et al., 2010; Becker et al., 2012; Shan et al., 2013; Jouanin et al., 2020). Fortunately, there were no adverse effects on gluten and dough qualities induced by the reduction or decrease of the gliadins content (Altenbach et al., 2014; Li et al., 2018). This may derive from the compensation effect from glutenin genes when gliadin genes are repressed or silenced (Becker et al., 2012; Li et al., 2018). There was a possible mechanism for this phenomenon: following the increase of glutenin, the glutenin/gliadin ratio was elevated; then more glutenin macropolymers were formed that could enhance the gluten strength and dough elasticity, therefore improving the breadmaking quality (Li et al., 2018). Consequently, the aim that simultaneous improvement of end-use and health benefits quality could realize by breeding the gliadin-free or reduced-gliadin wheat varieties. However, wheat varieties that have been genetically modified *via* RNAi and CRISPR/Cas9 still require ethics approval, which is a time-consuming process, before they can be commercially cultivated and included in food products. In the present study, 15 and 36 QTLs were revealed to be related to the total gliadin content and the contents of three gliadin fractions, respectively. Furthermore, three KASP markers were developed on the basis of the SNPs closely linked to QTL in 6AS-3, 6D-2, and 7DS-1. The utility of these KASP markers was confirmed in an RIL population and a set of wheat varieties collected worldwide. The findings of this study and the developed KASP markers will be useful for the artificial selection of the early generations during the breeding of reduced-gliadin wheat varieties.

## Data Availability Statement

The original contributions presented in the study are publicly available. This data can be found here: The RNA-seq data was available in Genome Sequence Archive (https://bigd.big.ac.cn/gsa/browse/CRA004223).

## Author Contributions

ZZ, ZL, ZW, and JH conceived and designed the experiments. SG and HG investigated the phenotype. CL, MQ, ZD, and WL conducted the field experiments. ZL and ZW supervised the study. ZZ, SG, and HG conducted statistical analysis of the phenotypic data and prepared the figures. SG, XS, and CL prepared the tables. ZZ and SG drafted the manuscript. ZZ, JH, and HG revised and edited the manuscript. All authors discussed the results, contributed to the manuscript, read, and approved the final manuscript.

## Conflict of Interest

The authors declare that the research was conducted in the absence of any commercial or financial relationships that could be construed as a potential conflict of interest.

## Publisher’s Note

All claims expressed in this article are solely those of the authors and do not necessarily represent those of their affiliated organizations, or those of the publisher, the editors and the reviewers. Any product that may be evaluated in this article, or claim that may be made by its manufacturer, is not guaranteed or endorsed by the publisher.

## Footnotes

1
http://plants.ensembl.org/index.html

## Supplementary Material

Supplementary Figure 1 QTL for the total gliadin content in the RIL population at two locations across 2 years. Gray horizontal lines indicate an LOD value of 2.5. The chromosomes that contain QTL with LOD >2.5 are indicated in red. E1, E2, E3, and E4 correspond to 2018–2019 in Yuanyang, 2018–2019 in Shangqiu, 2019–2020 in Yuanyang, and 2019–2020 in Shangqiu, respectively. BLUP indicates the QTL was detected according to the best linear unbiased prediction of gliadin contents.

Supplementary Figure 2 QTL for the ω-gliadin content in the RIL population at two locations across 2 years. Please refer to the Supplementary Figure 1 legend for more details.

Supplementary Figure 3 QTL for the α-gliadin content in the RIL population at two locations across 2 years. Please refer to the Supplementary Figure 1 legend for more details.

Supplementary Figure 4 QTL for the γ-gliadin content in the RIL population at two locations across 2 years. Please refer to the Supplementary Figure 1 legend for more details.

Supplementary Figure 5 Variations in the ω-gliadin content among different AX-108913293 genotypes in a set of wheat varieties collected worldwide. Phenotypic comparison of ω-gliadin contents among different genotypes detected using the KASP marker developed on the basis of AX-108913293 in 207 wheat varieties. E5, E6, and E7 correspond to 2017 in Yuanyang, 2018 in Yuanyang, and 2018 in Shangqiu, respectively. ^∗∗∗^ and ^∗∗^ indicate significant differences at the *P* < 0.001 and *P* < 0.01 levels, respectively.
