# Supplementary figures and images for: Dissection of the Genetic Architecture for Quantities of Gliadins Fractions in Wheat (Triticum aestivum L.)
Source: Front Plant Sci. 2022 Mar 24;13:826909. doi: 10.3389/fpls.2022.826909 (PMC8988047; doi:10.3389/fpls.2022.826909)

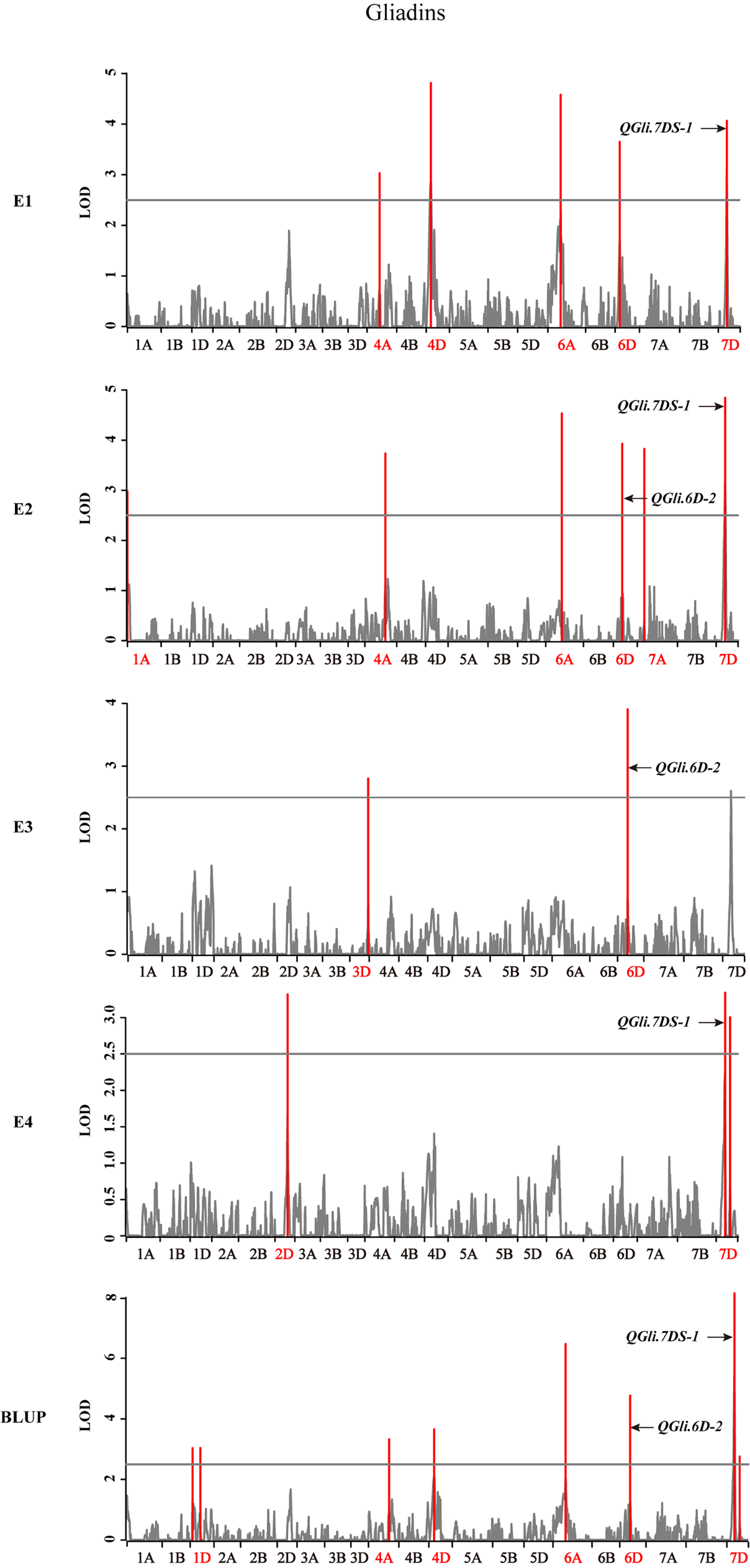

Supplement: Supplementary Figure 1 — QTL for the total gliadin content in the RIL population at two locations across 2 years. Gray horizontal lines indicate an LOD value of 2.5. The chromosomes that contain QTL with LOD >2.5 are indicated in red. E1, E2, E3, and E4 correspond to 2018–2019 in Yuanyang, 2018–2019 in Shangqiu, 2019–2020 in Yuanyang, and 2019–2020 in Shangqiu, respectively. BLUP indicates the QTL was detected according to the best linear unbiased prediction of gliadin contents. [file Image_1.TIF]

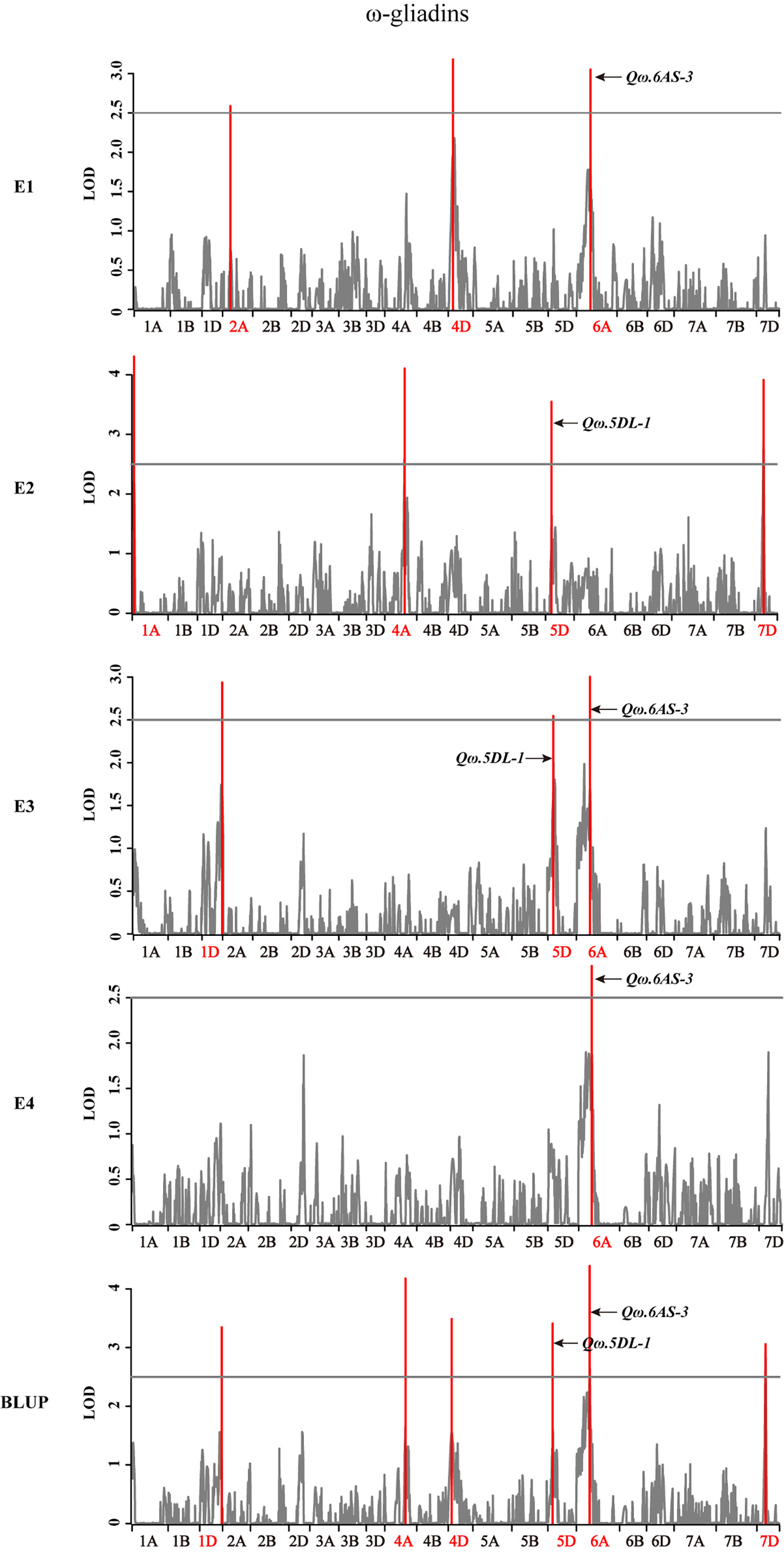

Supplement: Supplementary Figure 2 — QTL for the ω-gliadin content in the RIL population at two locations across 2 years. Please refer to the Supplementary Figure 1 legend for more details. [file Image_2.TIF]

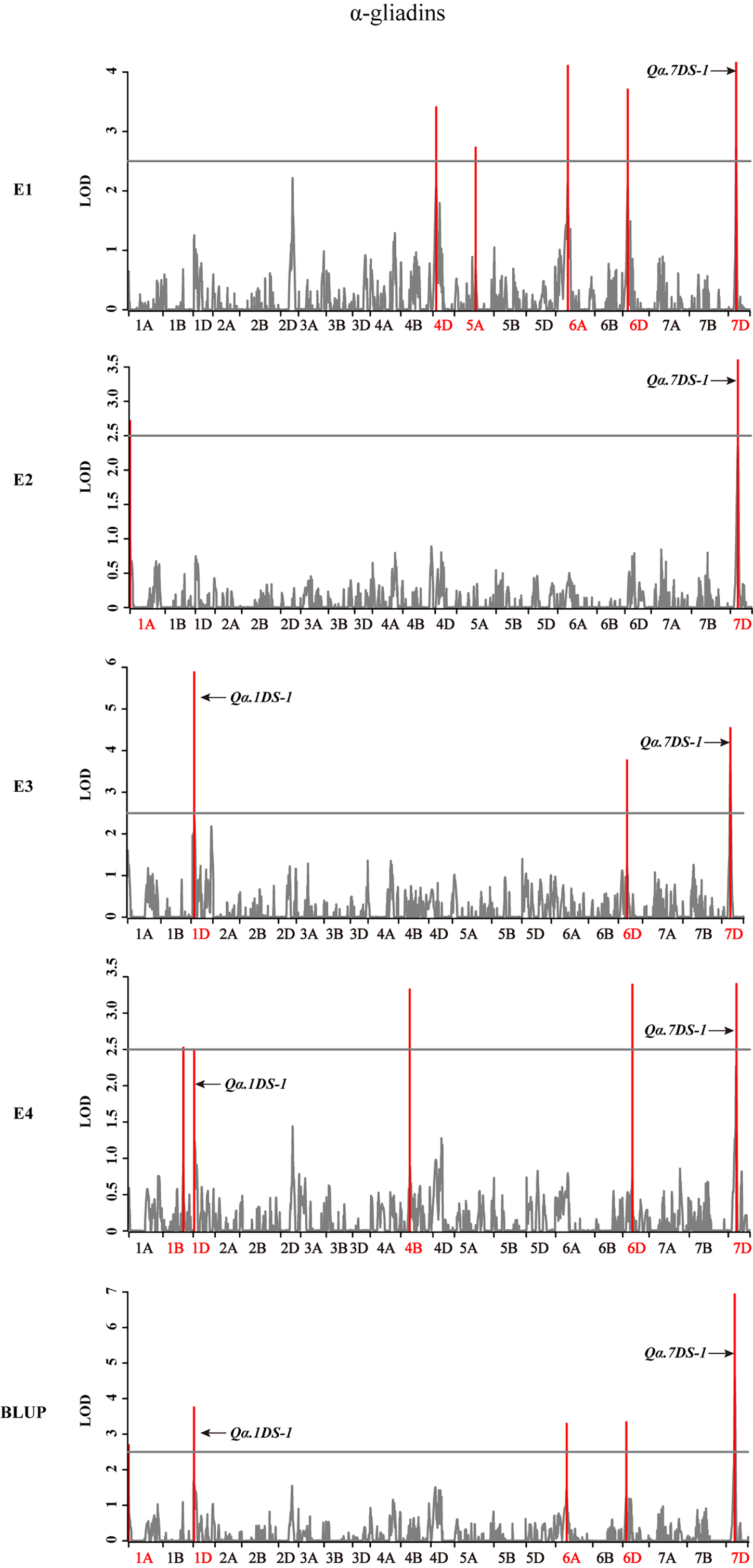

Supplement: Supplementary Figure 3 — QTL for the α-gliadin content in the RIL population at two locations across 2 years. Please refer to the Supplementary Figure 1 legend for more details. [file Image_3.TIF]

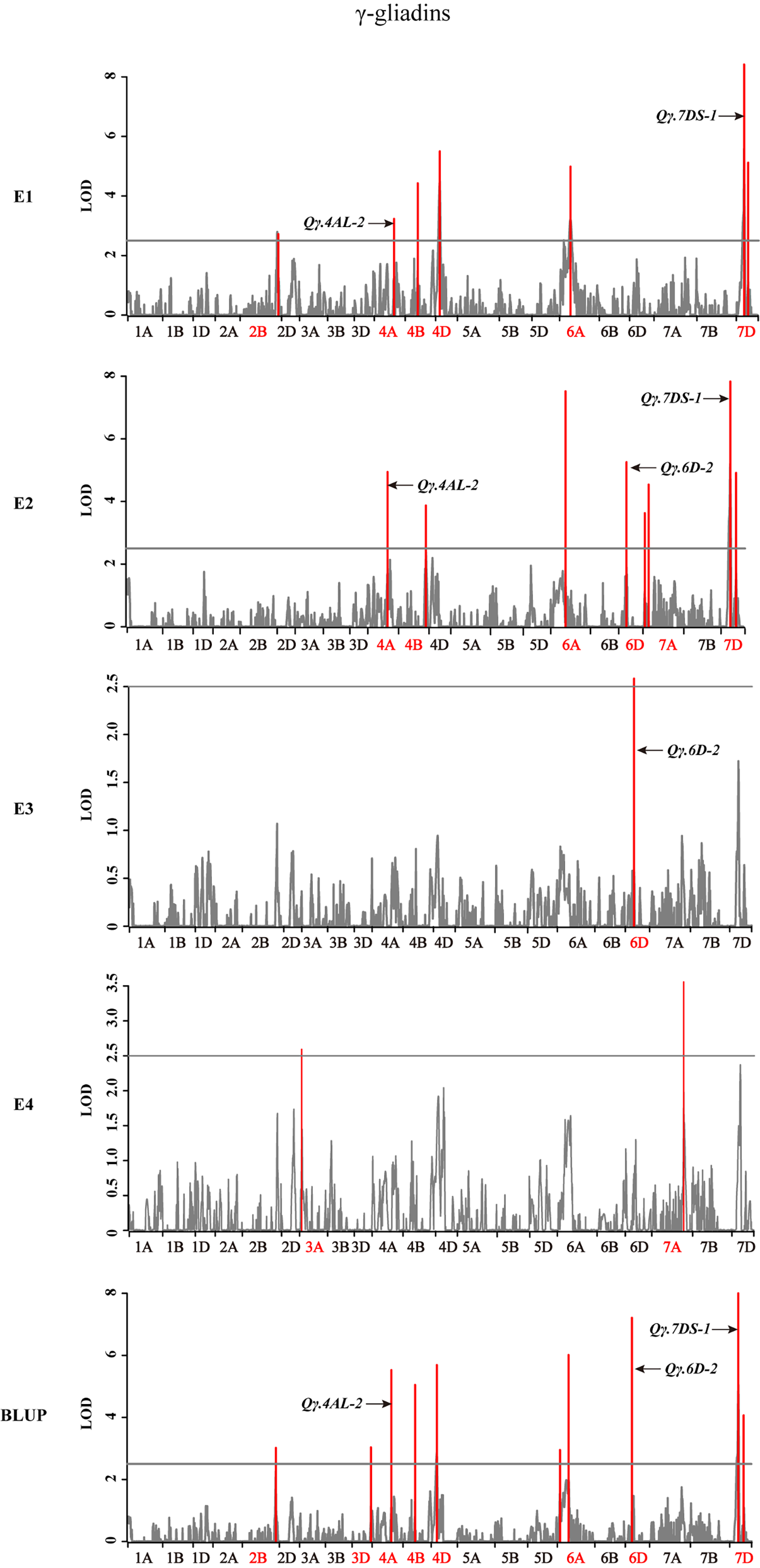

Supplement: Supplementary Figure 4 — QTL for the γ-gliadin content in the RIL population at two locations across 2 years. Please refer to the Supplementary Figure 1 legend for more details. [file Image_4.TIF]

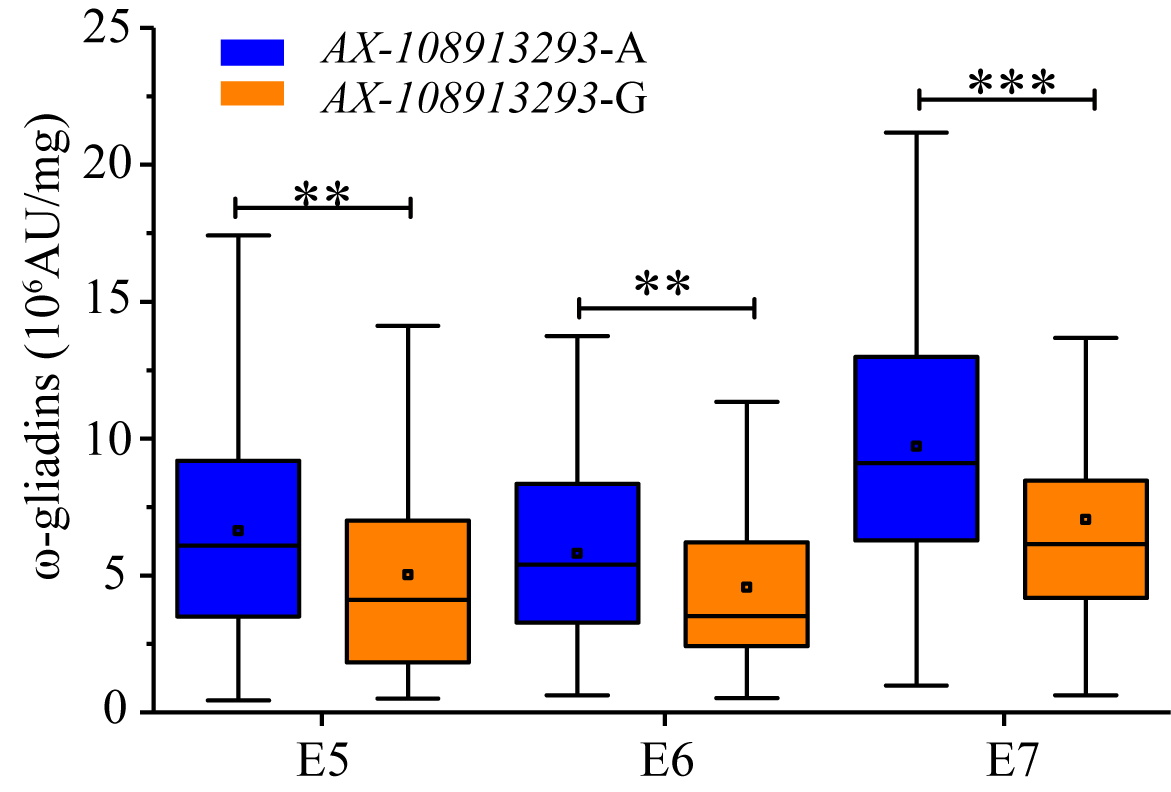

Supplement: Supplementary Figure 5 — Variations in the ω-gliadin content among different AX-108913293 genotypes in a set of wheat varieties collected worldwide. Phenotypic comparison of ω-gliadin contents among different genotypes detected using the KASP marker developed on the basis of AX-108913293 in 207 wheat varieties. E5, E6, and E7 correspond to 2017 in Yuanyang, 2018 in Yuanyang, and 2018 in Shangqiu, respectively. ∗∗∗ and ∗∗ indicate significant differences at the P < 0.001 and P < 0.01 levels, respectively. [file Image_5.TIF]
